# Low genetic diversity of *Ehrlichia canis* associated with high co-infection rates in *Rhipicephalus sanguineus* (*s.l.*)

**DOI:** 10.1186/s13071-018-3194-9

**Published:** 2019-01-07

**Authors:** Alejandro Cabezas-Cruz, Eleonore Allain, Abdullah S. Ahmad, Muhammad A. Saeed, Imran Rashid, Kamran Ashraf, Lena Yousfi, Wasim Shehzad, Lea Indjein, Manuel Rodriguez-Valle, Agustin Estrada-Peña, Dasiel Obregón, Abdul Jabbar, Sara Moutailler

**Affiliations:** 1 UMR BIPAR, INRA, ANSES, Ecole Nationale Vétérinaire d’Alfort, Université Paris-Est, 94700 Maisons-Alfort, France; 2 Department of Veterinary Biosciences, Faculty of Veterinary and Agricultural Sciences, The University of Melbourne, Werribee, Victoria 3030 Australia; 3 Department of Parasitology, Faculty of Veterinary Sciences, University of Veterinary and Animal Sciences, Lahore, Punjab Pakistan; 4 Institute of Biochemistry and Biotechnology, Faculty of Biosciences, University of Veterinary and Animal Sciences, Lahore, Punjab Pakistan; 5 Queensland Alliance for Agriculture & Food Innovation, The University of Queensland, St. Lucia, Queensland 4072 Australia; 6 Faculty of Veterinary Medicine, University of Zaragoza, Zaragoza, Spain; 7 Cell and Molecular Biology Laboratory, Center for Nuclear Energy in Agriculture, University of Sao Paulo, Piracicaba, SP Brazil

**Keywords:** *Rhipicephalus sanguineus* (*s.l.*), Co-infection, *Ehrlichia canis*, *Rickettsia massiliae*, Genetic diversity

## Abstract

**Background:**

*Rhipicephalus sanguineus sensu lato* (*s.l.*) is the most widely distributed ixodid tick and is a vector of major canine and human pathogens. High-throughput technologies have revealed that individual ticks carry a high diversity of pathogens, including bacteria, protozoa and viruses. Currently, it is accepted that co-infections (multiple pathogen species within an individual) are very common in ticks and influence pathogen acquisition and transmission as well as host infection risk. However, little is known on the impact of the genetic diversity of pathogens on the incidence of co-infections. Herein, we studied the frequency of co-infections in *R. sanguineus* (*s.l.*) and their association with the genetic diversity of *Ehrlichia canis*.

**Methods:**

*Rhipicephalus sanguineus* (*s.l.*) female ticks (*n* = 235) were collected from healthy farm dogs in three districts of Pakistan. Microfluidic real-time PCR, a powerful nanotechnology for high-throughput molecular detection of pathogens, was used to test the presence of 25 bacterial and seven parasitic species in individual ticks. The genetic diversity of *E. canis* was evaluated by characterizing the *trp36* gene.

**Results:**

A total of 204 ticks were infected with at least one pathogen and 109 co-infected with two (80%) or three (20%) pathogens. *Rickettsia massiliae* (human pathogen) and *E. canis* (zoonotic dog pathogen) were the most common pathogens co-infecting (30.4%) ticks. Furthermore, all identified co-infections included *R. massiliae* and/or *E. canis*. Multiple correspondence analysis (MCA) revealed that single infections did not show clear regional association whereas some co-infections were restricted to certain geographical regions. The sequence analysis of *trp36* in representative samples allowed the identification of three *E. canis* strains with low genetic diversity, and the strain found in Muzaffargarh district appeared to be more adapted to co-infection with *R. massiliae*.

**Conclusions:**

*Rhipicephalus sanguineus* (*s.l.*) harbors multiple co-infections with human and dog pathogens of zoonotic potential. Findings of this study suggest that genetic diversity of *E. canis* may favor co-infections with different pathogens.

## Background

Ticks (Acari: Ixodidae) are important ectoparasites of animals and humans that cause mechanical damage to their hosts and serve as vectors for the transmission of various pathogens of medical and veterinary importance [1–3]. Although tick-borne pathogens are maintained in stable natural cycles involving ticks and animals (domestic and/or wild), humans may serve as accidental hosts [4]. In the past few years, the distribution and abundance of many tick species have risen in canids worldwide, resulting in an increased prevalence of tick-borne pathogens in these animals [2, 5–7]. Given that domestic animals (such as dogs) live in close proximity to humans and they may act as reservoir hosts for pathogens that can infect humans, the distribution and dissemination of ticks in these animals could be a significant public health concern [8–10].

*Rhipicephalus sanguineus* (*sensu lato*), the brown dog tick, is the most widely distributed ixodid tick that infests human as well as canine dwellings [11]. Being a three-host tick, three different developmental stages (larvae, nymphs and adults) of *R. sanguineus* (*s.l.*) usually feed on dogs [1, 11]. *Rhipicephalus sanguineus* (*s.l.*) is known to transmit a number of pathogens that produce disease in dogs, including babesiosis (caused by *Babesia canis*, *B. gibsoni* and *B. vogeli*), ehrlichiosis (*Ehrlichia canis* and *Anaplasma platys*), hepatozoonosis (*Hepatozoon canis*), and rickettsioses/spotted fever (*Rickettsia rickettsii*, *R. massiliae* and *R. conorii*), especially in tropical and subtropical regions of the world [1, 11–15]. There are also reports of *R. typhi*, a rickettsia of the Typhus group mainly transmitted by fleas, detected in *R. sanguineus* (*s.l.*) [14]. Other important canine ticks (and pathogens they transmit) include *Amblyomma* spp. (*Hepatozoon* spp. and *E. canis*), *Dermacentor* spp. (*Babesia* spp., *Ehrlichia* spp. and *Rickettsia* spp.), *Haemaphysalis* spp. (*Babesia* spp. and *H. canis*), *Hyalomma* spp. (*Theileria annulata*), *Ixodes* spp. (*Borrelia* spp., *Ehrlichia* spp. and *Anaplasma* spp.) and *Rhipicephalus microplus* (*Anaplasma* spp.) in various parts of the world [3, 16–18]. Importantly, many of these tick-borne pathogens in dogs could be of zoonotic importance, which warrants for studies assessing tick-borne pathogens in ticks collected from dogs. The risk of zoonotic tick-borne diseases in dogs is especially important in geographical regions which offer conducive environments for the increasing of tick abundance [2, 6, 9].

Pakistan is an important agricultural country, located in South Asia (30°0' N, 70°0'E) and exhibits 10 different agro-ecological zones [6, 19, 20]. The vast majority of the country consists of sub-tropical and partially temperate regions which extend from Himalayas in the north to the Arabian Sea in the south [19]. Due to such favourable climatic conditions, a high prevalence of ticks and tick-borne diseases could be expected in these regions. Although limited data are available on canine ticks from Pakistan, *R. sanguineus* (*s.l.*) appears to be the most widespread canine tick species in this country, with prevalence rates as high as 98% in dogs [21–23]. Other ticks, including *Dermacentor* spp*.*, *Haemaphysalis* spp. and *Hyalomma* spp. have also been reported in dogs from Pakistan [21, 23]. Likewise, a high prevalence of tick-borne pathogens such as *Anaplasma* spp. and *Babesia* spp. have been observed in conventional diagnostic methods (e.g. blood smear examination) from dogs in Pakistan [21, 24–26]. Recent studies from Pakistan utilized molecular (*18S* rRNA gene amplification) approaches and reported a high prevalence (46% and 12%) of *H. canis* from farm and pet dogs, respectively [6, 27]. Overall, these studies indicate that multiple tick species and a variety of tick-borne pathogens are prevalent in dogs from Pakistan. However, there is paucity of information on the occurrence and magnitude of tick-borne pathogens and their co-infections in ticks collected from dogs in Pakistan. Co-infections of tick-borne pathogens have been reported to be common in several tick species in different geographical areas [28–30], including *R. sanguineus* (*s.l.*) [31–33], and dogs were found infected with more than one tick-borne pathogen [34–37]. This highlights the importance of considering co-infections in tick-borne pathogen surveys.

Given the spectrum of pathogens transmitted by ticks, some organisms may not be identified using artificial culture methods; hence, molecular approaches are indispensable for thorough detection of multiple tick-borne pathogens [2, 3, 38]. However, conventional molecular approaches (e.g. PCR) could amplify and detect only known target pathogens. Furthermore, only a restricted number of target pathogens could be amplified and tested due to limiting factors (e.g. quantity of DNA) in PCR. To address these issues, a high throughput epidemiological surveillance method (based on a microfluidic system) was developed [38, 39]. The system holds the capacity to perform parallel real-time PCR using a small volume/quantity of DNA and can process up to 9216 individual reactions simultaneously [38, 39]. This high throughput system has been used successfully for the detection of four tick-species and 37 tick-borne pathogens in Europe [38].

Very little is known about the tick-borne pathogens in canine ticks from Pakistan. Therefore, this study aimed to use the microfluidic high-throughput system to determine tick-borne pathogens of zoonotic importance in ticks collected from dogs in three different agro-ecological zones of Punjab, Pakistan.

## Methods

### Study area and tick samples

**Fig. 1:**
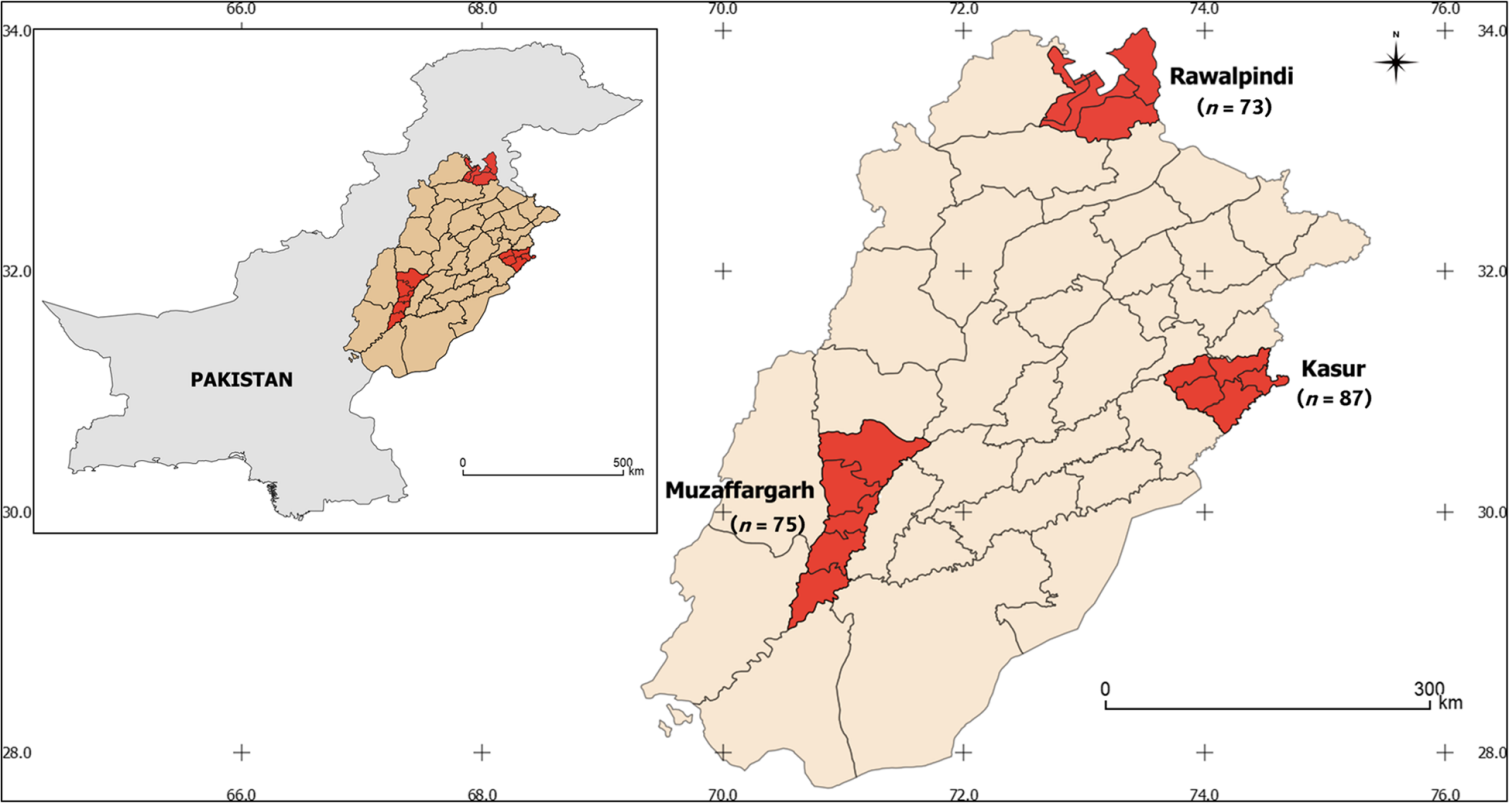
Map of Pakistan showing the sampling sites and the number of ticks tested per region

Single engorged adult female ticks (*n* = 235) were collected from clinically healthy farm dogs of both sexes (male, *n* = 70; female, *n* = 165) between June and October 2016 from three different districts in the province of Punjab, including Kasur (31°12'21"N, 74°45'81"E; *n* = 87), Muzaffargarh (30°07'36"N, 71°18'05"E; *n* = 75) and Rawalpindi (33°59'84"N, 73°04'41"E; *n* = 73) (Fig. 1). Following collection, each tick specimen was stored individually in 70% ethanol until used. For morphological identification, each tick was examined using a dissecting microscope (Olympus, Tokyo, Japan). Ticks were identified using the keys as described previously [40].

### DNA extraction and PCR pre-amplification

Prior to DNA extraction, ethanol was removed, and individual ticks were washed three times (30 min) in distilled H_2_O, and then ground using a plastic mortar. DNA was extracted using a DNeasy Blood and Tissue Kit (Qiagen, Hilden, Germany) following the manufacturer’s protocol. DNA was stored at -20 °C until further use.

For better detection of pathogen DNAs, total DNAs were pre-amplified with the Perfecta Preamp Supermix (Quanta Biosciences, Beverly, USA) according to the manufacturer’s instructions. Primers (targeted all pathogens) were pooled combining equal volume of primers (200 nM final each). The reaction was performed in a final volume of 5 μl containing 1 μl Perfecta Preamp 5X, 1.25 μl pooled primers mix, 1.5 μl distilled water and 1.25 μl DNA, with one cycle at 95 °C for 2 min, 14 cycles at 95 °C for 10 s and 60 °C for 3 min. At the end of the cycling program the reactions were diluted as 1:10. Pre-amplified DNAs were stored at -20 °C until further use.

### Microfluidic real-time PCR

To detect major tick-borne pathogens (see below), the BioMark^TM^ real-time PCR system (Fluidigm, California, USA) was used for high-throughput microfluidic real-time PCR amplification using the 48.48 dynamic arrays (Fluidigm). These chips dispensed 48 PCR mixes and 48 samples into individual wells, after which on-chip microfluidics assembled real-time PCR reactions in individual chambers prior to thermal cycling resulting in 2304 individual reactions. Targeted pathogens (and markers) included: *Borrelia* spp. (*23S*), *B. burgdorferi* (*rpoB*), *B. garinii* (*rpoB*), *B. afzelii* (*fla*), *B. valaisiana* (*ospA*), *B. lusitaniae* (*rpoB*), *B. spielmanii* (*fla*), *B. bissettii* (*rpoB*), *B. bissettii* (*glpQ*), *Anaplasma* spp. (*16S*), *A. marginale* (*msp1*), *A. platys* (*groEL*), *A. phagoctyophilum* (*msp2*), *A. ovis* (*msp4*), *A. centrale* (*groEL*), *A. bovis* (*groEL*), *Ehrlichia* spp. (*16S*), *Ehrlichia canis* (*gltA*), *Neorickettsia mikurensis* (*groEL*), *Rickettsia* spp. (*gltA*), *R. conorii* (ITS), *R. slovaca* (ITS), *R. massiliae* (ITS), *R. helvetica* (ITS), *R. helvetica* (ITS), *R. felis* (*orfB*), *Bartonella* spp. (*ssrA*), *B. henselae* (*pap31*), *Francisella tularensis* (*tu*l4 and *fopA*), *Coxiella* endosymbiont (*IS1111* and *icd*), *Apicomplexa* (*18S*), *Babesia microti* (*CCTeta*), *B. canis* (*hsp70*), *B. ovis* (*18S*), *B. bovis* (*CCTeta*), *B. caballi* (*rap*1), *Babesia* str. EU1 (*18S*), *B. divergens* (*hsp70*), *Mycoplasma* spp. (*16S*), *Theileria* spp. (*18S*) and *Hepatozoon* spp. (*18S*).

Briefly, amplifications were performed using 6-carboxyfluorescein (FAM)- and black hole quencher (BHQ1)-labeled TaqMan probes with TaqMan Gene expression master mix in accordance with manufacturer’s instructions (Applied Biosystems, Massachusetts, USA) [38]. PCR cycling comprised of a denaturation step at 95 °C for 5 min followed by 45 cycles at 95 °C for 10 s, 60 °C for 15 s and 40 °C for 10 s. One negative water control was included per chip. To assess inhibitory molecules (that could inhibit PCR) in samples, DNA from *Escherichia coli* (EDL933 strain) was added to each sample as an internal inhibition control, and primers and probe specific for the *eae* gene of *E. coli* were used.

### Validation of microfluidic real-time PCR results

Conventional and nested PCR using different primers than those of the BioMark^TM^ system were used to confirm the presence of the detected pathogens in the samples (Table 1). Amplicons were sequenced by Eurofins MWG Operon (Ebersberg, Germany) and assembled using the BioEdit software (Ibis Biosciences, Carlsbad, USA). An online BLAST (National Center for Biotechnology Information) was used to identify the sequenced organisms.

**Table 1:** Primers used for microfluidic real-time PCR

Species	Target gene	Forward primer (5'-3')	Reverse primer (5'-3')	Reference
*Babesia* spp., *Theileria* spp., *Hepatozoon* spp.	*18S* rRNA	GTGAAACTGCGAATGGCTCATTAC; GGCTCATTACAACAGTTATAGTTTATTTG	AAGTGATAAGGTTCACAAAACTTCCC; CGGTCCGAATAATTCACCGGAT	[66]
*Borrelia* spp.	*flaB*	GCAGTTCARTCAGGTAACGG; GCATCAACTGTRGTTGTAACATTAACAGG	GCAATCATAGCCATTGCAGATTGT; ACATATTCAGATGCAGACAGAGGT	[67]
*Anaplasma* spp., *Ehrlichia* spp.	*16S* rRNA	GAACGAACGCTGGCGGCAAGC; TGCATAGGAATCTACCTAGTAG	AGTAYCGRACCAGATAGCCGC; AGTAYCGRACCAGATAGCCGC	[68]
*Coxiella*-like endosymbiont	*16S* rRNA	CGTAGGAATCTACCTTRTAGWGG; TGAGAACTAGCTGTTGGRRAGT	ACTYYCCAACAGCTAGTTCTCA; GCCTACCCGCTTCTGGTACAATT	[69]
*Rickettsia* spp.	Citrate synthase	GGGGGCCTGCTCACGGCGG	ATTGCAAAAAGTACAGTGAACA	[70]

### Analysis of *Ehrlichia canis trp36* gene and encoded amino acid sequence

To assess the genetic diversity of *E. canis*, the full-length of the tandem repeat protein 36 (*trp36*) gene was amplified and sequenced. This gene codes for a major immunogenic protein and commonly used as a genetic marker to characterize the genetic diversity of this bacterium [41–43]. The amplification of *trp36* was performed using primers and following a protocol previously published [42]. The tandem repeats finder (TRF) database [44] was used to predict the presence of tandem repeats in the *trp36*. For sequence analysis, the predicted amino acid sequence of TRP36 was divided into three regions as previously reported [42, 43]. Region I, the 5' end pre-repeat region composed of 426–429 bp/142–143 aa at the N terminus of the encoded protein; Region II, the tandem repeat region (variable number of the 27 bp/9 aa repeat units depending on the strain); and Region III the 3' end post-repeat region (48, 84 and 90 bp/16, 28 and 30 aa) at the C terminus of the encoded protein.

### Phylogenetic analysis using *trp36*

Nucleotide sequences of the pre-repeat region of *E. canis trp36* were used for phylogenetic tree reconstruction as previously reported [41, 43]. Two sequences of the *trp36* ortholog in *E. chaffeensis* (*trp47*) were used as the outgroup. Sequences available in the GenBank database were collected and aligned on MAFFT software [45], configured for the highest accuracy. The best-fit model of sequence evolution was selected based on Corrected Akaike Information Criterion (cAIC) and Bayesian Information Criterion (BIC) as implemented in MEGA v.6.00 [46]. Tamura’s 3-parameter (T92) [47] model of amino acid evolution, which had the lowest values of cAIC and BIC, was chosen for tree reconstruction. The phylogenetic tree was reconstructed using the Maximum Likelihood (ML) method in MEGA. Initial tree for the heuristic search was obtained automatically by applying Nearest-Neighbor-Join and BioNJ algorithms to a matrix of pairwise distances estimated using Maximum Composite Likelihood (MCL), and then selecting the topology with superior log-likelihood value. The analysis involved 30 nucleotide sequences. All positions containing gaps and missing data were eliminated. Reliability of internal branches was assessed using the bootstrapping method (1000 replicates). Graphical representation and editing of the phylogenetic trees were performed in MEGA.

### Statistical analysis

Chi-square test was used to evaluate the co-occurrence of bacteria and to compare prevalence among different regions. Student’s t-test was used to assess significant differences of multiple co-infections in ticks according to sites of collection. Data were analyzed using GraphPad 5 Prism program (GraphPad Software Inc., La Jolla, CA, USA). Multiple correspondence analysis (MCA) was used to analyze the pattern of relationships between single and co-infections and tick origin. To this aim, a standard ‘Burt matrix’ analysis was performed. All possible pairwise pathogen combinations were calculated as (a)(a-1)/2 and all possible triple pathogen combinations were calculated as (a)(a-2)/3, where ‘a’ is the number of individual pathogen(s).

## Results

### Prevalence of pathogens and *Coxiella* endosymbiont

A total of 204 (of 235, 87%) ticks were infected with at least one pathogen. *Rickettsia massiliae* was the most commonly (68%) detected pathogen followed by *E. canis* (51%) and *Anaplasma* spp. (17%), including *A. phagocytophilum* (6%), *A. marginale* (4%), *A. centrale* (3%), *A. platys* (3%) and *A. ovis* (1%). Furthermore, *Coxiella*-like endosymbionts (hereafter *Coxiella*-like) and *B. canis* were detected in 5% and 3% of samples, respectively. Only a few pathogens were identified as single infections, including *R. massiliae* (24.3%), *E. canis* (12.8%), *A. platys* (0.9%), *A. phagocytophilum* (1.7%) and *A. marginale* (0.9%), respectively (Table 2).

**Table 2:** Prevalence of pathogens in ticks collected from farm dogs in three districts of Pakistan

Pathogen	Kasur (*n*)	Muzaffargarh (*n*)	Rawalpindi (*n*)	Total (*n*)	%
Single infections					
*R. massiliae*	25	21	11	57	24.3
*E. canis*	6	1	23	30	12.8
*Coxiella*-like	0	0	0	0	0
*B. canis*	0	0	0	0	0
*A. platys*	0	2	0	2	0.9
*A. phagocytophilum*	4	0	0	4	1.7
*A. ovis*	0	0	0	0	0
*A. marginale*	1	0	1	2	0.9
*A. centrale*	0	0	0	0	0
Double co-infections					
*R. massiliae* + *E. canis*	8	38	16	62	26.4
*R. massiliae* + *Coxiella*-like	0	2	0	2	0.9
*R. massiliae* + *A. marginale*	3	0	0	3	1.3
*R. massiliae* + *A. phagocytophilum*	5	0	0	5	2.1
*E. canis* + *B. canis*	3	0	0	3	1.3
*R. massiliae* + *A. centrale*	3	0	1	4	1.7
*R. massiliae* + *B. canis*	2	0	0	2	0.9
*R. massiliae* + *A. platys*	2	0	0	2	0.9
*E. canis* + *A. phagocytophilum*	1	0	1	2	0.9
*E. canis* + *A. centrale*	0	0	1	1	0.4
*R. massiliae* + *A. ovis*	0	0	1	1	0.4
Triple co-infections					
*R. massiliae* + *E. canis* + *Coxiella*-like	1	6	2	9	3.8
*R. massiliae* + *E. canis* + *A. marginale*	1	0	3	4	1.7
*R. massiliae* + *E. canis* + *B. canis*	0	3	0	3	1.3
*R. massiliae* + *E. canis* + *A. phagocytophilum*	1	1	0	2	0.9
*R. massiliae* + *E. canis* + *. platys*	2	0	0	2	0.9
*R. massiliae* + *E. canis* + *A. ovis*	0	0	0	0	0
*R. massiliae* + *E. canis* + *A. centrale*	0	0	1	1	0.4
*E. canis* + *A. centrale* + *Coxiella*-like	0	0	1	1	0.4

**Fig. 2:**
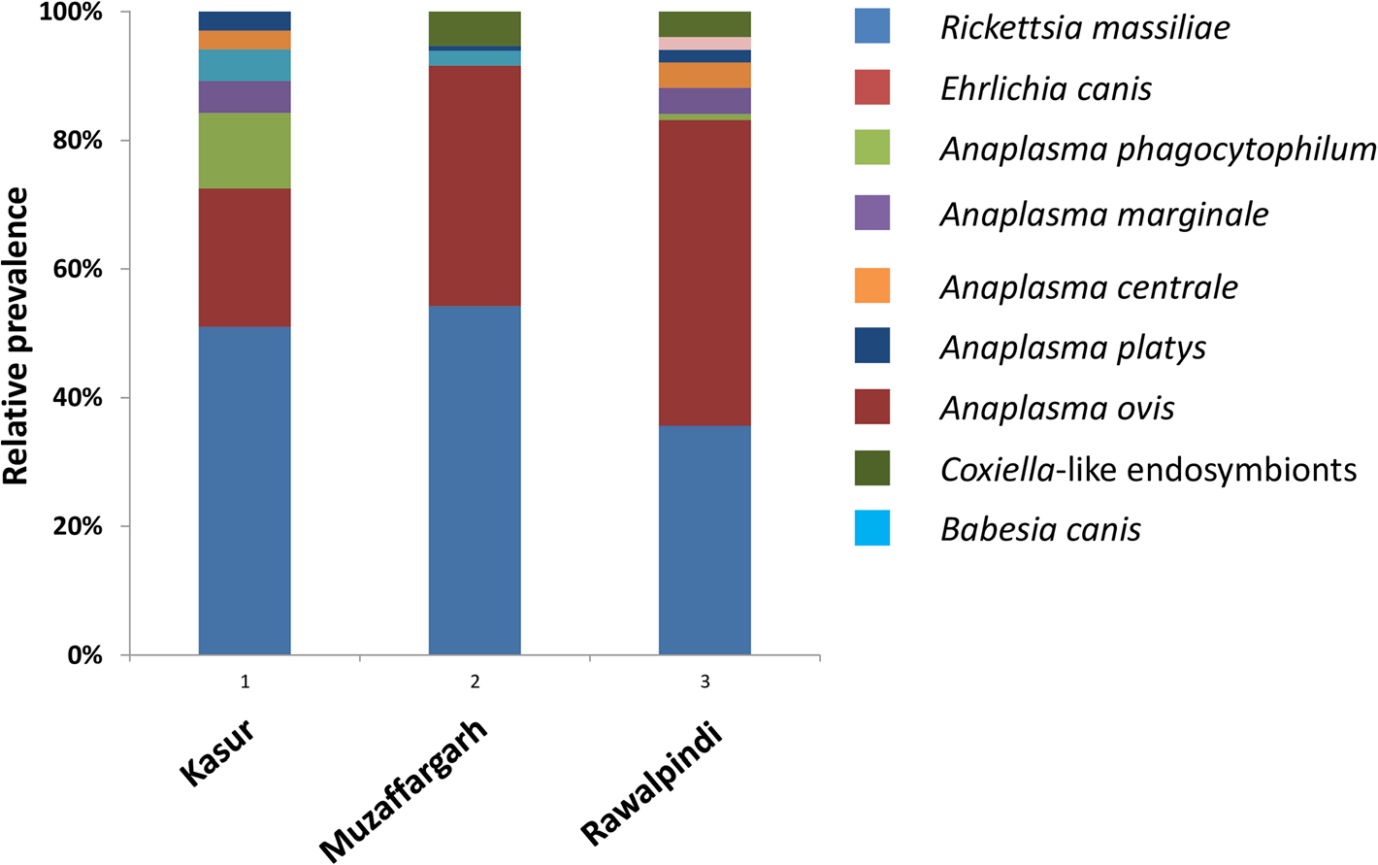
The district-wise relative prevalence of individual pathogens and *Coxiella*-like endosymbiont detected from *Rhipicephalus sanguineus* (*s.l.*) in Pakistan

Based on location, the highest prevalence (of mono-specific infection) was found in Muzaffargarh (98%) followed by Rawalpindi (84%) and Kasur (78%). The prevalence was significantly different between Muzaffargarh and Kasur (*χ*^2^ = 18.9, *P* < 0.0001), and Rawalpindi and Muzaffargarh (*χ*^2^ = 12.0, *P* < 0.003). The region with the lowest microorganism diversity was Muzaffargarh where only five of the nine microorganisms were detected (Fig. 2). *Rickettsia massiliae*, *E. canis* and *A. platys* were detected in all three regions studied. However, *Coxiella*-like was detected only in Muzaffargarh and Rawalpindi. Likewise, *A. phagocytophilum*, *A. marginale* and *A. centrale* were found in Kasur and Rawalpindi only (Fig. 2). The prevalence of *E. canis* was significantly different between Muzaffargarh and Kasur (*χ*^2^ = 4.7, *P* < 0.05), Rawalpindi and Muzaffargarh (*χ*^2^ = 34.9, *P* < 0.0001), and Rawalpindi and Kasur (*χ*^2^ = 19.9, *P* < 0.0001), although no differences were found for *R. massiliae* among regions.

### Co-infections of ticks

Among the 204 infected ticks, 108 (53%) were found to be co-infected with two (80%) or three (20%) pathogens. Some microorganisms (e.g. *Coxiella*-like, *B. canis*, *A. ovis* and *A. centrale*) were found only in mixed infections (Table 2). The most frequent co-infection was due to *R. massiliae* and *E. canis* with a prevalence of 26.4% (*P* < 0.005). All other co-infections had prevalence values lower than 5% which were not statistically significant.

Of 21 possible triple pathogen combinations included in the assay, only eight were detected in this study although all without any significant associations (Table 2). All triple co-infections included both *R. massiliae* and *E. canis* except for one co-infection (*E. canis*, *A. centrale* and *Coxiella*-like) with a very low prevalence (Table 2). A significant association (*P* < 0.05) was observed between *E. canis* and *R. massiliae* in both Muzaffargarh and Rawalpindi districts. The prevalence of *R. massiliae* + *E. canis* co-infection was significantly different between Muzaffargarh and Kasur (*χ*^2^ = 38.1, *P* < 0.0001) and between Rawalpindi and Muzaffargarh (*χ*^2^ = 17.01, *P* < 0.0003).

**Fig. 3:**
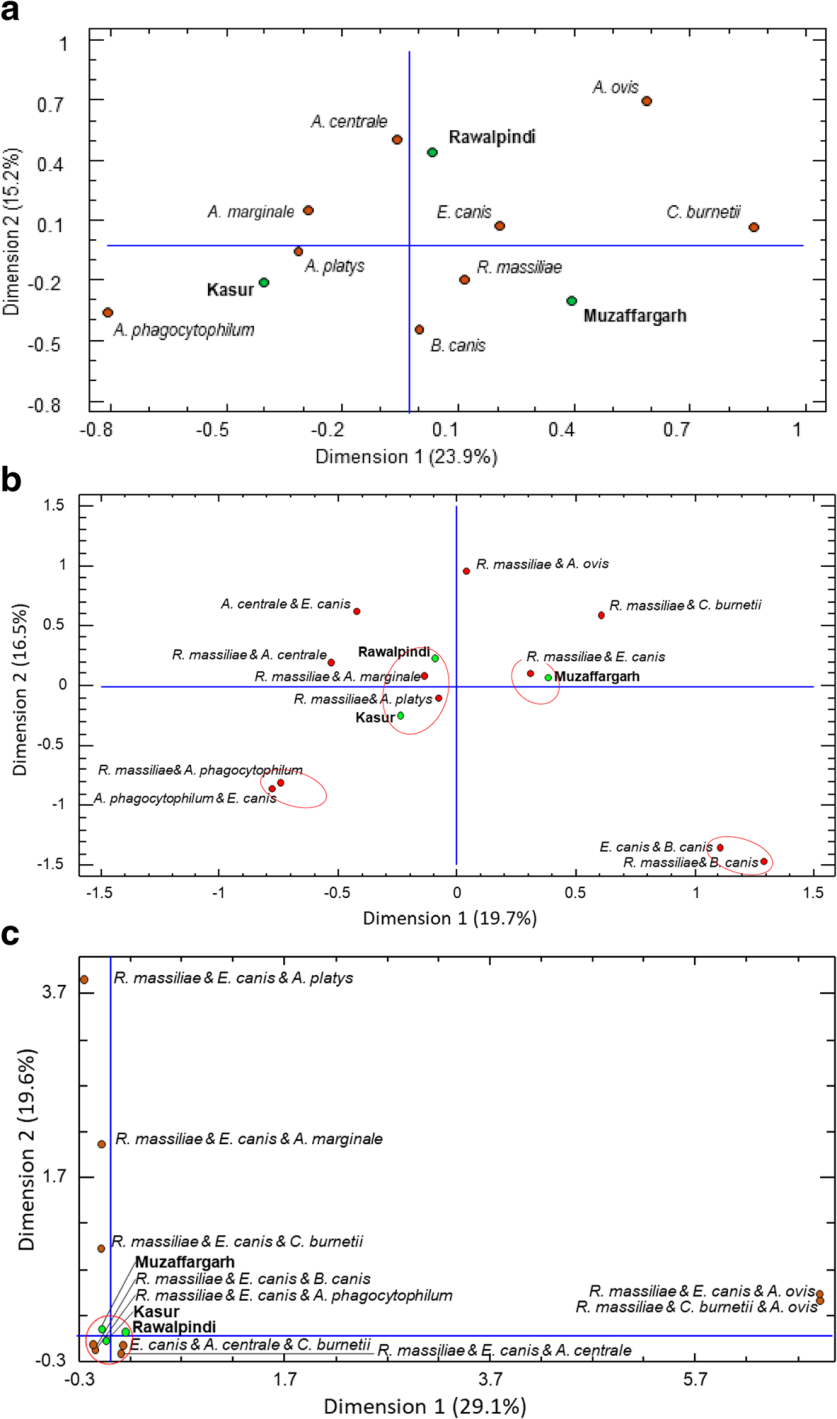
Multiple correspondence analyses showing associations between regions (green dots) and pathogen(s) detected (red dots) in *Rhipicephalus sanguineus* (*s.l.*) ticks. Clusters of associations are shown to facilitate interpretation as well as spatial distribution. Analyses are shown for single infections (**a**), and double (**b**) and triple (**c**) co-infections

Multiple correspondence analysis was performed to further test correlation between infections and geographic regions. When only a single pathogen (regardless of being part of a co-infection) was considered, the MCA confirmed the variation in the presence and prevalence of single pathogens among various agroclimatic regions (Fig. 3a). When double and triple co-infections were considered, the MCA revealed associations between regions and co-infections. Based on double co-infection analyses, Kasur and Rawalpindi clustered together with co-infections, including *R. massiliae* + *A. platys* and *R. massiliae* + *A. marginale*, respectively (Fig. 3b). Muzaffargarh clustered separately and harbored co-infection of *R. massiliae* + *E. canis*. In addition, two pairs of co-infections, including *A. phagocytophilum* (i.e. *R. massiliae* + *A. phagocytophilum* and *E. canis* + *A. phagocytophilum*) and *B. canis* (i.e. *E. canis* + *B. canis* and *R. massiliae* + *B. canis*) clustered irrespective of any particular region (Fig. 3b). When triple infections were considered, Kasur, Rawalpindi and Muzaffargarh lied in the center of the multidimensional matrix, and around these points the clustered triple infections were of *R. massiliae* + *E. canis* + *B. canis*, and *R. massiliae* + *E. canis* + *A. phagocytophilum*, *E. canis* + *A. centrale* + *Coxiella*-like, and *R. massiliae* + *E. canis* + *A. centrale*, indicating an independent distribution of triple infections in three regions (Fig. 3c). In addition, a strong correspondence was observed between the triple infections *R. massiliae* + *E. canis* + *A. ovis* and *R. massiliae* + *Coxiella*-like + *A. ovis*, which also showed the highest values of inertia (less independence).

### Genetic variability of *Ehrlichia canis*

The full coding region of the gene *trp36* was amplified and sequenced in four *E. canis*-positive samples per region (*n* = 12). Following theoretical translation of all *trp36* nucleotide sequences into proteins, three different sequences were identified and named Kasur (GenBank: MH608289), Muzaffargarh (GenBank: MH608290) and Rawalpindi (GenBank: MH549197). To test the genetic diversity of *E. canis* in ticks from Pakistan, we performed three types of analyses based on *trp36* nucleotide and protein sequences as described previously [43], including (i) nucleotide and amino acid sequences identity; (ii) molecular signatures of the pre-repeat region of TRP36 (i.e. presence/absence of glycine (G) 117 and sequence of putative glycosylation sequon at asparagine (N) 125; (iii) tandem repeat composition of TRP36; (iv) length of post-repeat region; and (v) phylogenetic analysis using pre-repeat region of the gene *trp36*.

**Fig. 4:**
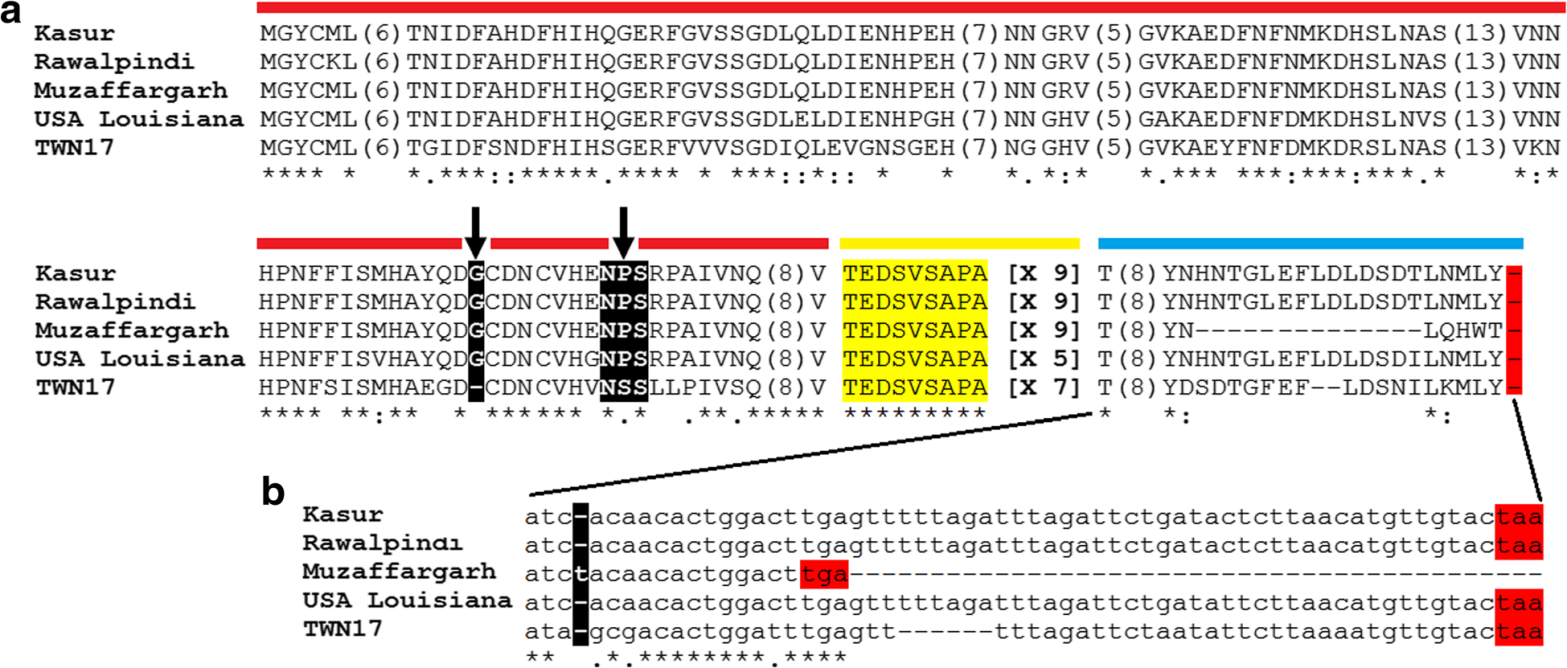
*Ehrlichia canis* strain analysis based on *trp36* sequences. **a** TRP36 amino acid sequences of the three different strains of *E. canis* identified in Pakistan (Kasur, Rawalpindi and Muzaffargarh) were aligned with TRP36 sequences of *E. canis* strains previously reported with low (USA Louisiana) and high (TWN17) genetic diversity. For sequence analysis, TRP36 is separated in three regions: pre-repeat (red), tandem repeat (yellow) and post-repeat (blue). Molecular signatures (presence of G at position 117 and the sequence of the putative N-glycosylation sequon at N125, NPS/NSS) in the pre-tandem region are highlighted in black boxes. The numbers of tandem repeats per strain are shown in square brackets. The sequence identical and similar amino acid positions are indicated with asterisks and dots, respectively. For figure simplification purpose, large stretches of regions with 100% identity were removed from the figure and the amino acid length of these regions is shown in parentheses. **b** *trp36* nucleotide sequences showing the insertion of thymine (t, black box) in Muzaffargarh strain. Stop codons are highlighted in red boxes

The *E. canis trp36* nucleotide and putative protein sequences reported in this study shared more than 99% and 97% identity at the nucleotide and amino acid levels, respectively (Table 3). The amino acid sequence variation of Pakistan strains relative to those from the USA (Louisiana) and Taiwan (TWN17) is displayed in Fig. 4a. These were included as representatives of low and high genetic diversity strains, respectively [41, 43]. The pre-repeat region of TRP36 amino acid sequences from Pakistan had molecular signatures of previously reported *E. canis* strains of low genetic diversity (Fig. 4a). Particularly, the presence of G at position 117 and the sequence of the putative glycosylation sequon at N125 was NPS (arrows in Fig. 4a). The tandem repeat region of the three TRP36 sequences obtained in this study contained 9 repetitions of the sequence ‘TEDSVSAPA’ (highlighted in yellow in Fig. 4a). Unexpectedly, the *trp36* sequences from Muzaffargarh and Kasur were 100% identical at the nucleotide level but shared only 97.9% identity at the amino acid level (Table 3). Multiple sequence alignment revealed that the Muzaffargarh *trp36* sequence had an insertion of thymine (t) at position 707 (Fig. 4b). This insertion shifted the open reading frame of *trp36* which resulted in amino acid changes in the post-repeat region (Fig. 4a) and an early stop codon ‘TGA’ (Fig. 4b). As a result, while the post-repeat region in sequences from Kasur and Rawalpindi was 30 amino acids long, the post-repeat region in that from Muzaffargarh was only 16 amino acids long. Variable sizes (16, 28 and 30 amino acids) in the post-repeat regions of TRP36 have been described previously [41, 43].

**Table 3:** Percentage of identity of *trp36* nucleotide (above the diagonal) and putative amino acid sequences (below the diagonal) between *Ehrlichia canis* strains

*E. canis* strain	GenBank ID	Kasur	Rawalpindi	Muzaffargarh	USA Louisiana	TWN17
Kasur	MH608289	–	99.87	100	98.48	91.76
Rawalpindi	MH549197	99.60	–	99.86	98.29	91.44
Muzaffargarh	MH608290	97.92	97.45	–	98.53	91.58
USA Louisiana	DQ146151	95.41	94.84	93.14	–	90.57
TWN17	HQ009756	84.98	84.21	84.62	82.33	–

**Fig. 5:**
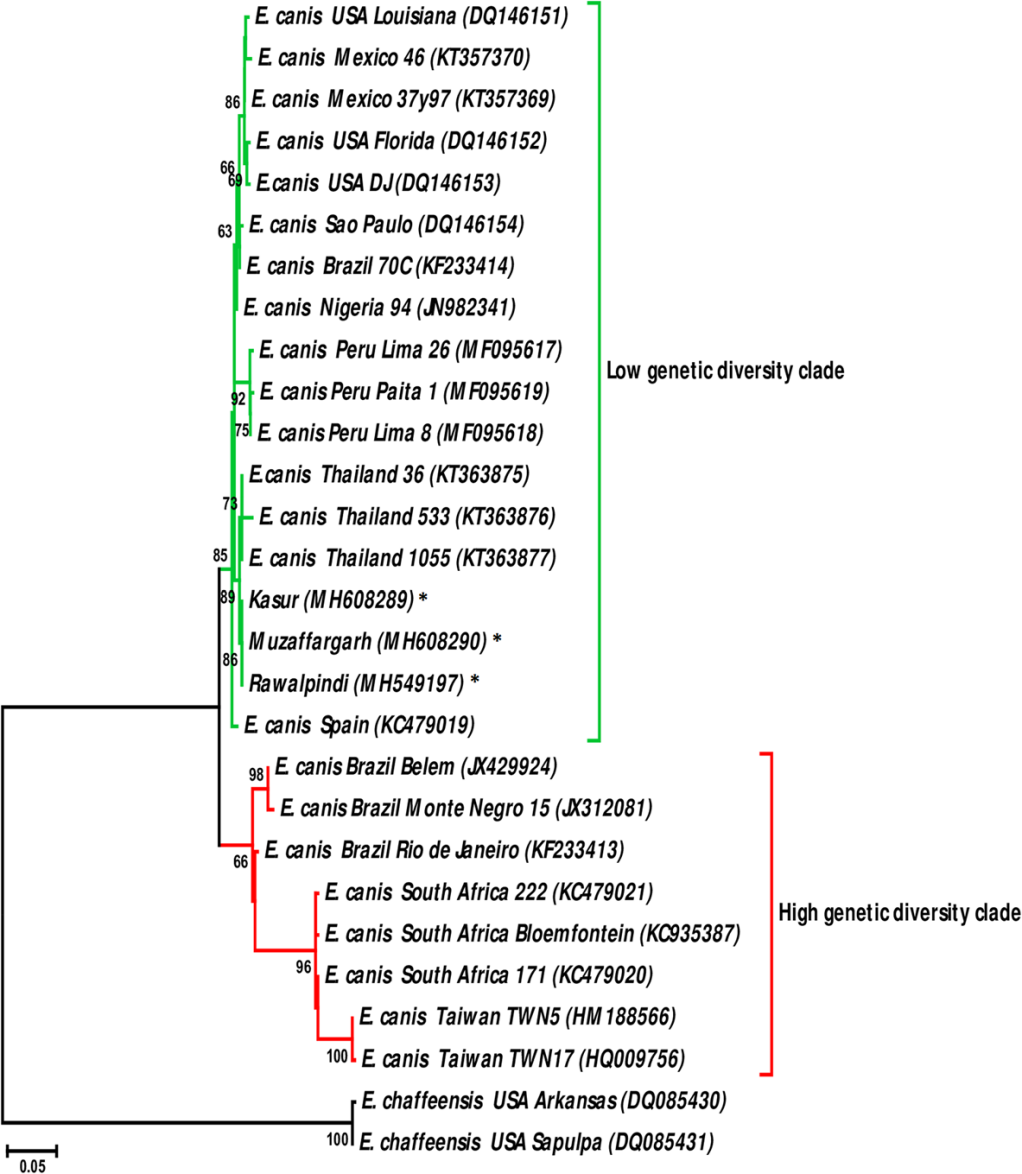
Phylogenetic analysis of *Ehrlichia canis trp36* sequences identified in Pakistan and other regions of the world. The figure shows the maximum likelihood phylogenetic tree inferred with nucleotide sequences of the pre-repeat region of *trp36*. Strains from Pakistan are labeled with asterisks. *Ehrlichia chaffeensis trp47*, the ortholog of *trp36*, sequences were used as the outgroup. The analysis involved 28 nucleotide sequences. All positions containing gaps and missing data were eliminated. There were a total of 387 positions in the final dataset. Reliability of internal branches was assessed using the bootstrapping method (1000 replicates)

A previous study reported that *trp36* grouped *E. canis* strains in two well-defined phylogenetic clusters, i.e. one containing highly variable strains and the other with similar ones [41]. A maximum likelihood phylogenetic analysis was then performed to test the phylogenetic position of *E. canis* strains from Pakistan. In agreement with the molecular signatures described above, Fig. 5 shows that the *E. canis* strains from Pakistan clustered in the low genetic diversity clade.

Because each region had one specific *E. canis* strain, and ticks in each region had different prevalence of single infection with *E. canis* and co-infection with *R. massiliae* and *E. canis*, we hypothesized that different *E. canis* strain may have different fitness for single infection or co-infection with *R. massiliae* between regions. To test this hypothesis, the prevalence of *E. canis* strains in single infections and co-infections with *R. massiliae* was compared between regions using Chi-square test (Table 4). Results showed that the prevalence of co-infection with *E. canis* and *R. massiliae* in Muzaffargarh was significantly higher than mono-infection of *E. canis* in Kasur and Rawalpindi.

**Table 4:** Comparison of *Ehrlichia canis* strains prevalence in single infections and co-infections with *Rickettsia massiliae*

*E. canis* strain	Single infection *n* (%)	Co-infection *n* (%)	Total *n*	Chi-square analysis	
				Regional combinations	
Kasur	6 (43)	8 (57)	14	Kasur *vs* Muzaffargarh	*χ*^2^ = 45.2, *P* < 0.0001
Muzaffargarh	1 (3)	38 (97)	39	Kasur *vs* Rawalpindi	*χ*^2^ = 5.1, *P* = 0.07
Rawalpindi	23 (59)	16 (41)	39	Muzaffargarh *vs* Rawalpindi	*χ*^2^ = 73.3, *P* < 0.0001

## Discussion

To our knowledge, this is the first comprehensive study on genetic characterization of pathogens in *R. sanguineus* (*s.l.*) ticks collected from farm dogs in three different agro-climatic regions of Punjab, Pakistan. *Rhipicephalus sanguineus* (*s.l.*) is known to transmit a number of pathogens that produce diseases in dogs and humans, especially in tropical and subtropical regions of the world [1, 11–15]. However, there is paucity of information on the prevalence of pathogens in *R. sanguineus* (*s.l.*) from dogs in Asia. Although, standard PCR is most frequently used tool for the detection of tick-borne pathogens, it could be biased as pathogen detection is strongly influenced by particular research interests [48]. Microfluidic real-time PCR (a high-throughput state-of-the-art technology) presents an alternative solution as it has the ability to detect a diverse array of tick-borne pathogens [28, 38]. Therefore, we used microfluidic real-time PCR approach to test the presence of 25 bacterial and seven parasite species in individual ticks. Furthermore, the genetic diversity of the most commonly detected pathogen (*E. canis*) was assessed using the *trp36* gene sequencing.

*Rhipicephalus sanguineus* (*s.l.*) harbored multiple co-infections with human and dog pathogens of zoonotic potential. Importantly, all the ticks used for this study were partially or fully engorged and therefore the detection of pathogen DNA either represents the most recent blood meal and/or a prior infection of the tick. Once acquired by a tick, tick-borne pathogens are transmitted transstadially and thus, each blood meal increases the chance of acquiring new pathogens which in turn increases the likelihood of co-infections within the tick. This is supported by several field surveys in which co-infections have been reported in several tick species, including *Ixodes ricinus* [28, 49], *I. scapularis* [29, 50], *Dermacentor reticulatus* [30], *Haemaphysalis longicornis* [33] and *R. sanguineus* (*s.l.*) [31–33].

An important finding in this study is the detection of a wide array of pathogen combinations in *R. sanguineus* (*s.l.*) ticks from Pakistan, potentially due to the use of high-throughput approach (Table 2). For instance, only eight different combinations of tick-borne pathogens in *I. ricinus* have been reported in previous studies using standard serological and molecular (PCR) approaches [49, 51–55]. However, 31 different tick-borne pathogen combinations were detected in the same tick species using microfluidic real-time PCR, indicating that this technique could produce superior results than the standard PCR [28]. We identified 19 different pathogen combinations occurring in *R. sanguineus* (*s.l.*) from Pakistan (Table 2). However, only nine pathogen combinations have been reported in *R. sanguineus* (*s.l.*) in previous studies, including *Ehrlichia* spp., *Anaplasma* spp., *Hepatozoon* spp., *Babesia* spp., *Leishmania* spp. and *Cercopithifilaria* spp. [31–33]. Importantly, our assay included not only pathogens commonly identified in *R. sanguineus* (*s.l.*) (e.g. *E. canis*) [56] but also pathogens rarely found in this tick species (e.g. *Borrelia* spp.) [33].

*Ehrlichia canis* was found in vast majority (12/19; 63%) of co-infections in *R. sanguineus* (*s.l.*), which is in agreement with previous studies [31, 33]. In addition, our results also showed a ubiquitous presence (15/19; 79%) of *R. massiliae* in the identified co-infections. Whether *E. canis* and/or *R. massiliae* facilitate the acquisition and transmission of other pathogens in *R. sanguineus* (*s.l.*) remains elusive, although at least one of these pathogens was found in all co-infections. Further studies are required in this context as co-infections could considerably impact the epidemiology of tick-borne pathogens. For instance, recent studies have indicated that *Babesia microti* (the causative agent of human babesiosis) is emerging in areas endemic for *Borrelia burgdorferi* (the causative agent of Lyme disease) in the USA [57]. The emergence of *Babesia microti* has become difficult to explain because this pathogen has a low ecological fitness characterized by poor transmission from *Peromyscus leucopus* mice to larval ticks and poor transstadial transmission from larvae to nymphs [57]. The current hypothesis, supported by empirical data, is that *B. burgdorferi* increases *B. microti* transmission from *Peromyscus leucopus* mice, which act as reservoirs for both *B. microti* and *B. burgdorferi* (*s.l.*) to ticks [57]. Interestingly, a recent study using *Pseudomonas aeruginosa* and five different phage virus parasites as a model showed that co-infections can accelerate host adaptation and diversification [58]. Considering the findings in [58], it can be hypothesized that infection with multiple bacteria species may accelerate ecological innovation in ticks with a potential impact in tick fitness and pathogen transmission [59].

Since, the genetic diversity of tick-borne bacterial pathogen impacts pathogenicity, virulence, host specificity, prevalence and transmission [48, 60, 61], we hypothesize that it could also influence co-infections. The effect of strain diversity on tick-borne pathogen co-infection remains poorly studied, although co-infection with multiple strains of *A. marginale* is well-documented in ticks and hosts [60, 61]. Our study did not specifically evaluate the effect of genetic diversity of *E. canis* on co-infection within individual ticks. However, all the samples from the same region contained an identical *E. canis trp36* sequence which is suggestive of local adaptation of *E. canis* strains. Remarkably, the strain Muzaffargarh appeared to be more adapted or permissive to co-infection with *R. massiliae*. Some strains of *Flavobacterium columnare*, an environmental opportunistic bacterium, were found to be more permissible to co-infections in *Danio rerio* [62]. In co-infection systems, interacting pathogens can compete, cooperate or coexist [63]. The high rate of co-infection between *E. canis* strain Muzaffargarh and *R. massiliae* and the low rate of single infection of *E. canis* strain Muzaffargarh suggest that this strain has very low fitness in single infections and/or that it cooperates effectively with *R. massiliae* to infect ticks.

Previous studies indicated that *trp36* sequences of *E. canis* strains were the most variable among other immunodominant protein-encoding genes (e.g. *gp200*, *gp140*, *gp19*) sequenced [64]. Thus, small nucleotide variation in *trp36* indicates low genetic diversity in *E. canis*. We found low genetic diversity of *E. canis* in the three regions of Pakistan. Similar results were reported in Taiwan where all identified strains were highly similar between them [42]. Low genetic diversity of *E. canis* appears to be associated with high prevalence of this pathogen in ticks which contrasts with results obtained with *A. marginale* where high prevalence was associated with high genetic diversity of these bacteria in cattle. Despite low genetic diversity, *trp36* sequences differences allowed the identification of three distinct *E. canis* strains (one from each region) which grouped under the same clade. Intrinsic transmission efficiency of specific *E. canis* strains may explain strain predominance in regions of high prevalence. Collectively, these data suggest a minor strain variation and vast geographical spread of the bacterial parasite in Pakistan. It is possible that these genotypes might have diverged within the country and have not been introduced as such from other countries. However, intercontinental movement of dogs could also be a contributory factor in the spread of *R. sanguineus* (*s.l.*) and associated pathogens [23, 65].

## Conclusions

This study reports that tick-borne pathogen co-infections are very common in *R. sanguineus* (*s.l.*) ticks from Pakistan. The high prevalence of co-infection with *E. canis* and *R. massiliae* in *R. sanguineus* (*s.l.*) suggests a synergism between the two bacterial pathogens which in turn potentially increases the likelihood of acquiring a third pathogen. Low genetic diversity of *E. canis* was associated with high prevalence of this bacterium in *R. sanguineus* (*s.l.*) of Pakistan. The strain *E. canis* Muzaffargarh seems to be more adapted than others to co-infection with *R. massiliae* in *R. sanguineus* (*s.l.*)
